# *Dnali1* is required for sperm motility and male fertility in mice

**DOI:** 10.1186/s12610-023-00205-y

**Published:** 2023-11-23

**Authors:** Yiling Zhou, Yaling Wang, Jingwen Chen, Bangguo Wu, Shuyan Tang, Feng Zhang, Chunyu Liu, Lingbo Wang

**Affiliations:** Shanghai Key Laboratory of Metabolic Remodeling and Health, Institute of Metabolism and Integrative Biology, Institute of Reproduction and Development, Obstetrics and Gynecology Hospital, Fudan University, Shanghai, 200433 China

**Keywords:** Infertility, Sperm motility, Asthenozoospermia, Sperm flagellum, ICSI

## Abstract

**Background:**

The sperm flagellum is an evolutionarily conserved specialized organelle responsible for sperm motility and male fertility. Deleterious mutations in genes involved in the sperm flagellum assembly can often cause sperm motility defects and male infertility. The murine *Dnali1* gene encodes a protein that is known to interact with the cytoplasmic dynein heavy chain 1.

**Results:**

A *Dnali1*-mutated mouse model was generated by inducing a nonsense mutation in the *Dnali1* gene. The *Dnali1*-mutated male mice presented impaired sperm motility and were completely infertile. Although no obviously abnormal sperm morphology was observed in *Dnali1*-mutated male mice, the ultrastructural structure of sperm flagellum was disrupted, displaying as an asymmetrical distribution of the longitudinal columns (LCs). Notably, infertile *Dnali1*-mutated male mice were able to obtain offspring via ICSI.

**Conclusions:**

Our results uncover a role of DNALI1 in sperm motility and male fertility in mice, and demonstrate that ICSI overcomes *Dnali1*-associated male infertility, thus providing guidance for the diagnosis and genetic counseling of *DNALI1*-associated human infertility.

**Supplementary Information:**

The online version contains supplementary material available at 10.1186/s12610-023-00205-y.

## Résumé

**Contexte:**

Le flagelle des spermatozoïdes est un organite spécialisé conservé au cours de l’évolution, responsable de la mobilité des spermatozoïdes et de la fertilité mâle. Les mutations délétères des gènes impliqués dans l’assemblage du flagelle des spermatozoïdes peuvent souvent causer des défauts de mobilité des spermatozoïdes et une infertilité mâle. Le gène murin Dnali1 code pour une protéine flagellaire connue pour interagir avec la chaîne lourde 1 de la dynéine cytoplasmique.

**Résultats:**

Un modèle murin muté au niveau de Dnali1 a été généré par induction d’une mutation non-sens dans le gène Dnali1. Les souris mâles mutées au niveau de Dnali1 présentaient une altération de la mobilité des spermatozoïdes et étaient complètement infertiles. Bien qu’aucune morphologie manifestement anormale des spermatozoïdes n’ait été observée chez les souris mâles mutées au niveau de Dnali1, l’ultrastructure du flagelle des spermatozoïdes est perturbée, se présentant avec une distribution asymétrique des colonnes longitudinales. En particulier, les souris mâles infertiles mutées au niveau de Dnali1 ont pu obtenir une progéniture au moyen de l’injection intracytoplasmique de spermatozoïdes.

**Conclusions:**

Nos résultats révèlent un rôle de DNALI1 dans la mobilité des spermatozoïdes et dans la fertilité mâle chez la souris; ils montrent que l’ICSI surmonte l’infertilité mâle associée à Dnali1, fournissant ainsi des conseils pour le diagnostic et le conseil génétique de l’infertilité masculine associée à DNALI1.

**Mots-clés:**

Infertilité; Mobilité des Spermatozoïdes; Asthénozoospermie; Flagelle des Spermatozoïdes; ICSI.

## Introduction

The sperm flagellum is an axoneme-based structure which is essential for sperm motility [1]. As the core structure of sperm flagellum, the axoneme consists of highly ordered “9 + 2” microtubules characterized by a central microtubule pair connected to nine peripheral outer microtubule doublets (MTDs) [2, 3]. Radial spokes that link peripheral doublets to the central microtubule pair, nexin-dynein regulatory complexes that connect each peripheral doublet, and dynein arms that are multiprotein ATPase complexes, are also essential components for the axoneme [4, 5]. Among them, dynein arms, including the inner and the outer dynein arm (IDA and ODA) within the axoneme, provide the motor apparatus for the movement of the sperm flagellum through ATP hydrolysis, and are indispensable for flagellar beating [6, 7].

Peri-axonemal structures surrounding the axoneme, such as outer dense fibers (ODFs), mitochondrial sheath (MS), and fibrous sheaths (FSs), are required for structural cohesion, energy regulation, or cell signaling [1, 8, 9]. According to the location of peri-axonemal structures on the sperm flagellum, the sperm flagellum can be subdivided into four segments: connecting piece, midpiece, principal piece, and end piece [10]. The MS is located on the midpiece, and the FS is located on the principal piece; while the ODF surrounds the axoneme in both the midpiece and principal piece [1]. However, the distribution and structure of ODFs differ between the midpiece and principal piece of sperm flagella. In the midpiece, ODFs contain nine fibers, each of which is associated with a microtubule doublet; while in the principal piece, the third and eighth ODFs are replaced by two longitudinal columns (LCs) of the FS, with corresponding diminished fibers ending at the annulus [11].

The murine dynein axonemal light intermediate chain 1 (*Dnali1*) gene is localized on chromosome 4 and consists of six exons. It is enriched in spermatocytes, spermatids, and sperm flagella, indicating a potential function in spermatogenesis [12]. The orthologue gene in *Tetrahymena thermophila* is known as *p28*, and a previous study revealed a role of *p28* in ciliary motility [13]. Recently, two studies reported that mutations in *DNALI1* gene is associated with human infertility [14, 15]. In mice, two *Dnali1* knockout mouse lines were reported recently, including one line with a large deletion (6307 bp) covering exons 3–5 of murine *Dnali1* [15], and the other line with a conditional knockout of *Dnali1* in male germ cells [16]. Both lines displayed disrupted sperm motility and abnormal flagellum ultrastructure. Here, by generating and analyzing a mouse model carrying a nonsense mutation in exon 5 of the murine *Dnali1* gene, we clarify the functions of DNALI1 in sperm motility and sperm flagellum assembly, and provides experimental evidence that the assisted reproduction by ICSI overcomes *Dnali1*-associated male infertility.

## Materials and methods

### Generation of *Dnali1*-mutated mouse model

A nonsense mutation was induced in the exon 5 of murine *Dnali1* gene (*i.e.* conversion of a “CAG” codon to a “TAG” STOP-codon) using zygotic base-editing-mediated iSTOP-technology [17]. Briefly, the single-guide RNA (sgRNA) was specifically designed against the exon 5 of murine *Dnali1* (target: TGAACAGAAGGCGAAATGCG). sgRNA and CBE mRNA used for zygotic injection were in vitro transcribed according to the instructions as previously reported [17]. Injected zygotes were cultured to the two-cell embryo stage, followed by embryo transfer into foster female mice (8 weeks old, ICR) to obtain founder mice. Female founder mice were crossed with WT C57BL/6 mice to establish the mutant mouse line. The nonsense mutation (“TAG” STOP-codon) in *Dnali1* was verified in founder mice and their offspring through PCR and Sanger sequencing. All animal experiments were carried out in accordance with the recommendations of the US National Institutes of Health’s Guide for the Care and Use of Laboratory Animals, and the ethical guidelines of Fudan University. The primers used are presented in Table S1.

### Real-time quantitative PCR and reverse transcription PCR

Total RNA of mouse testes was extracted using the AllPrep DNA/RNA/Protein Mini Kits (80004, QIAGEN) or Trizol reagent (15596018, Invitrogen), and converted into cDNAs by a Hiscript III 1st Stand cDNA Synthesis Kit (R312-02, Vazyme). The obtained cDNAs were individually diluted tenfold to be used as templates for the subsequent real-time quantitative PCR with ChamQ SYBR qPCR Master Mix (Q311-03, Vazyme). *Gapdh* was used as an internal control. The expression of mRNA was quantified according to the 2^−ΔΔCt^ method.

### Immunoblotting analysis

The proteins of mouse tissues were extracted using RIPA (P0013B, Beyotime) or AllPrep DNA/RNA/Protein Mini Kits (80004, QIAGEN), then denatured at 95 ℃ for 10 min. The denatured proteins were separated on 10% SDS–polyacrylamide gels and transferred to a polyvinylidene difluoride (PVDF) membrane (IPVH00010, Millipore) for the immunoblotting analysis. After a blocking step in 5% milk diluted with TBST (TBS-0.1% Tween 20) for 1 h, the membranes were incubated overnight at 4 ℃ using the anti-DNALI1 (1:750, 17601–1-AP, Proteintech, or 1:1000, ab155490, Abcam), anti-GAPDH (1:3000; abs132004, Absin) or anti-α-TUBULIN (1:1500; PTM-5442, PTM BIO) antibodies diluted in blocking buffer. Then the membranes were washed in TBST and incubated with HRP conjugated secondary antibody (1:2500; SA00001-2, Proteintech) diluted in blocking solution for 1 h at room temperature. After washing three times in TBST, blots were revealed using the ECL reagents (WBKLS0100, Sigma-Aldrich) and a chemiluminescent imaging system (4600, Tanon).

### Analyses of sperm morphology, sperm motility and flagellar ultrastructure

Hematoxylin and eosin (H&E) staining was conducted to assess sperm morphology. For sperm count and sperm motility analysis, the fresh sperm samples of WT and *Dnali1*^−/−^ adult male mice were released from the cauda epididymis into 1 mL of HTF capacitation solution (MR-070-D, Millipore), and incubated at 37 ℃ for 15 min. Sperm characteristics of mice were evaluated using the computer-assisted sperm analysis system. Transmission electron microscopy (TEM) assays were performed on sperm to analyze flagellar ultrastructure. For TEM, the prepared sperm were washed and immersed in 2.5% phosphate buffered glutaraldehyde, washed three times with 0.1 mol/L phosphate buffer (PB, pH = 7.2), and post fixed with 1% osmium tetroxide in 0.1 mol/L PB at 4 ℃ for 1–1.5 h. Dehydration was conducted using graded ethanol (50%, 70%, 90%, and 100%) and 100% acetone, followed by infiltration with 1:1 acetone and SPI-Chem resin overnight at 37 ℃. After infiltration and embedding in Epon 812, the specimens were sliced with ultra-microtome and stained with uranyl acetate and lead citrate. The samples were observed and photographed via a TEM (TECNAI-10, Philips) with an accelerating voltage of 80 kV.

### Histological analysis of tissues

Fresh tissues were obtained from WT and *Dnali1*^−/−^ adult male mice, fixed in Bouin’s solution (HT10132, Sigma-Aldrich) or 4% PFA, embedded in paraffin, and sectioned to a 5-μm thickness. After deparaffinization and rehydration, sections were stained with hematoxylin–eosin (G1120, Solarbio Science & Technology) or Periodic acid-Schiff (PAS) (G1281, Solarbio Science & Technology).

### Immunofluorescence (IF)

For IF, cryosections of mouse testes were rinsed in PBS, permeabilized with 0.5% Triton X-100, and treated with 10% goat serum (AR1009, Boster Biological Technology). Then the slides were incubated overnight at 4 °C with anti-DNALI1 (1:150; 17601–1-AP, Proteintech) antibody. After washing three times with PBS, the slides were incubated with Alexa Fluor 568 conjugated secondary antibody (1:1000; A11036, Thermo Fisher) for 1h, and counterstained with Hoechst 33342 (1:1000; H1399, Thermo Fisher) at room temperature for 10 min. Fluorescence images were collected using a laser scanning confocal microscope (Nikon A1, Nikon). Sperm were washed in PBS, fixed in 4% PFA for 30 min at room temperature, and coated on adhesion microscope slides (80312–3161-16, CITOGLAS). Then the slides of sperm smears were washed in 1 mL of PBS and blocked in 10% donkey serum before being incubated overnight at 4 ℃ with the following primary antibodies: rabbit polyclonal anti-DNAH2 (1:100; HPA067103, Sigma-Aldrich), anti-DNAH17 (1:100; a gift from Professor Qinghua Shi to Dr. Chunyu Liu), monoclonal mouse anti-α-TUBULIN (1:500; T9026, Sigma-Aldrich). Washes were performed using PBS with 0.1% (v/v) Tween-20, followed by incubation at room temperature for 1 h with secondary antibodies Alexa Fluor 488 anti-Mouse IgG (1:1000; A32766, Invitrogen) and Cy3-conjugated AffiniPure Goat anti-Rabbit IgG (1:4000; 111–165-003, Jackson). Images were captured with a confocal microscope (LSM 880, Zeiss).

### Intracytoplasmic sperm injection (ICSI)

B6D2F1 (C57BL/6 × DBA2) female mice (8 to 10 weeks old) were superovulated as previously described [18]. In brief, the female mice were superovulated by injecting 7.5 IU of pregnant mare serum gonadotropin, followed by 7.5 IU of human chorionic gonadotropin 48h later. For ICSI, oocytes were obtained from superovulated female mice, and sperm heads were injected into oocytes as previously described [19–21]. The reconstructed embryos were cultured in KSOM medium (MR-107-D, Millipore) at 37 ℃ under 5% CO_2_ to the two-cell stage, and then were transferred into the oviducts of pseudopregnant foster female mice (8 weeks old, ICR) at 0.5 day postcoitum.

### Statistics

At least three biological replicates were used for all data presented with statistical analyses. Evaluation of statistical significance between two groups was performed with unpaired Student’s *t*-tests.* P* values are defined as follows: * *P* < 0.05; ** *P* < 0.01; ***P < 0.001; not significant (ns) as *P* > 0.05.

## Results

### Absence of the evolutionarily conserved DNALI1 protein leads to male infertility in mice

**Fig. 1:**
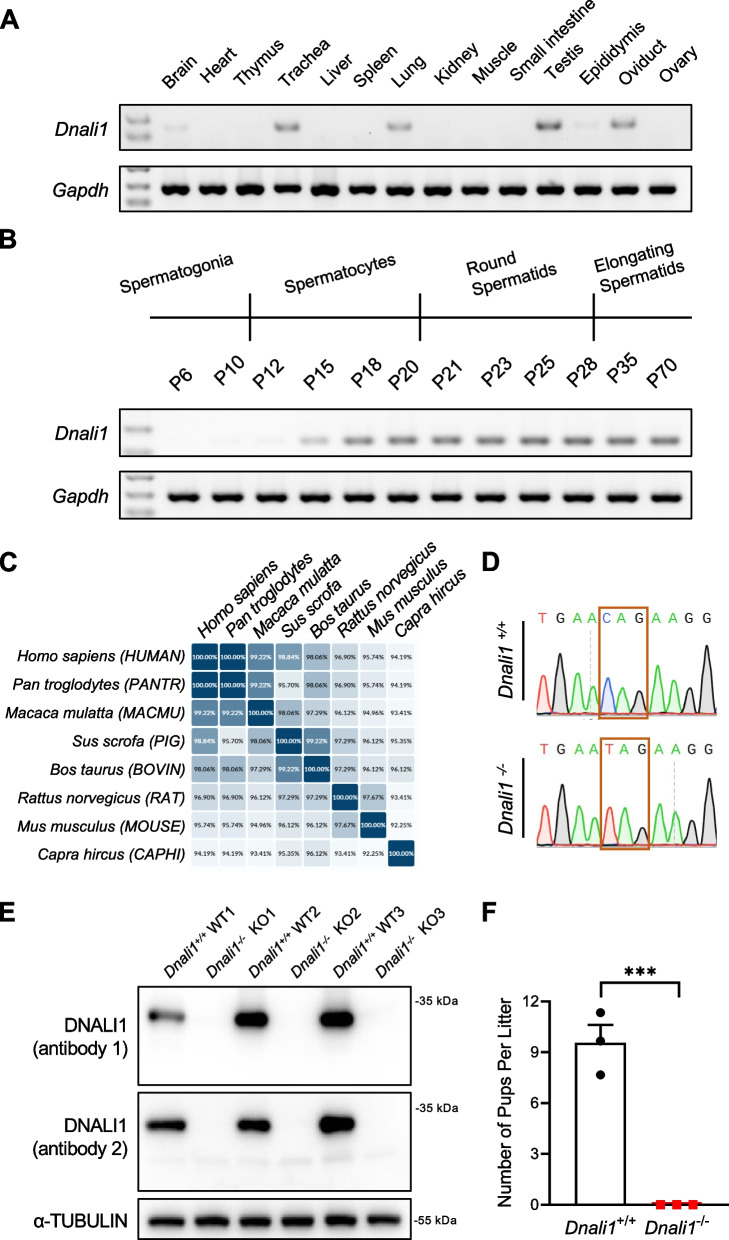
*Dnali1* is a conserved and testis-enriched gene whose deficiency causes male infertility. **A** Gene expression levels of *Dnali1* in the indicated WT mouse tissues. DNA marker was shown in the first lane. **B** Gene expression analysis showing *Dnali1* mRNA levels in the developing postnatal WT mouse testes. *Gapdh* was used as the internal control. DNA marker was shown in the first lane. **C** An interspecies protein alignment from the Uniport database, showing high conservation of DNALI1 in mammals. **D** Sequencing chromatograms of *Dnali1*^+/+^ and *Dnali1*^−/−^ mouse gDNA showing the intended nonsense mutation in the *Dnali1*^−/−^ mouse. **E** Immunoblotting showing the absence of DNALI1 protein (with no truncated protein) in the testis of *Dnali1*^−/−^ male mice. Two types of antibodies against DNALI1 were used (Antibody 1: Proteintech-17601–1-AP; Antibody 2: Abcam-ab155490). *n* = 3 mice for both the *Dnali1*^+/+^ and *Dnali1*^−/−^ groups respectively. α-TUBULIN was used as a loading control. **F** Fecundity testing of adult *Dnali1*^+/+^ and *Dnali1*^−/−^ male mice during a 3-month observation window. The average litter size was calculated by dividing total numbers of pups by the total numbers of litters. ***P < 0.001, unpaired Student’s *t*-test. WT: Wild-type

To investigate the physiological functions of *Dnali1*, we first examined its expression levels across various tissues obtained from WT mice, and found that *Dnali1* expression was highly enriched in the testis (Fig. 1A). Further *Dnali1* transcript level in WT mouse testes from different postnatal days was determined. During postnatal spermatogenesis, *Dnali1* expression was initiated at approximately postnatal day 15 (P15); and then the expression of *Dnali1* increased and remained elevated from postnatal day 18 (P18) (Fig. 1B). An interspecies protein sequence alignment indicated that DNALI1 is highly conserved in mammals (Fig. 1C).

To characterize the function of *Dnali1* in spermatogenesis, we further constructed *Dnali1*-mutated mice. Sanger sequencing of the PCR-amplicon from the targeted region confirmed the presence of a nonsense mutation in *Dnali1*-mutated mice, which causes premature translational termination of the DNALI1 protein (Fig. 1D). Real-time quantitative PCR (qPCR) assays (with primers listed in Table S1) verified a dramatically reduced mRNA expression of *Dnali1* in the testes from *Dnali1* homozygous mutated (*Dnali1*^−/−^) male mice compared to the WT control (Fig. S1). Immunoblotting assay showed an absence of DNALI1 protein in tissues of *Dnali1*^−/−^ male mice (Fig. 1E and Fig. S2). To explore the effect of the *Dnali1* deficiency on fertility, WT and *Dnali1*^−/−^ male mice (10 weeks of age) were mated with adult WT females, and the numbers of pups per litter were counted. We found that *Dnali1*^−/−^ male mice failed to produce any offspring after mating with WT adult female mice during a three-month observation window (Fig. 1F).

### *Dnali1* deficiency causes damaged sperm motility

**Fig. 2:**
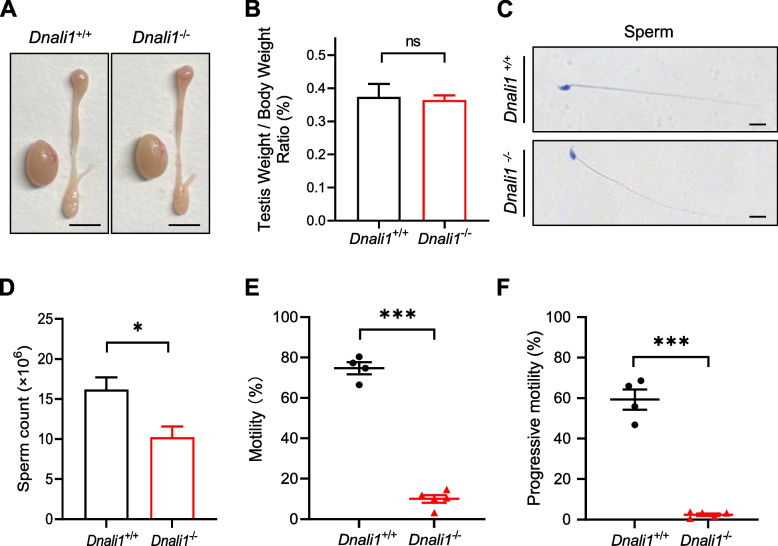
*Dnali1* deficiency leads to damaged sperm motility. **A** Gross morphology of testes and epididymides obtained from adult *Dnali1*^+/+^ and *Dnali1*^−/−^ mice. Scale bar, 5 mm. **B** The ratio of testis weight/body weight of *Dnali1*^+/+^ and *Dnali1*^−/−^ male mice. **C** H&E staining of sperm from the cauda epididymides of *Dnali1*^+/+^ and *Dnali1*^−/−^ male mice. Scale bar, 10 μm. (**D**) CASA analysis of the sperm counts in *Dnali1*^+/+^ and *Dnali1*^−/−^ male mice. (**E–F**) CASA analyses of the sperm motility (**E**) and progressive motility (**F**) in *Dnali1*^+/+^ and *Dnali1*^−/−^ male mice. ns (not significant): *P* > 0.05, *P < 0.05, ***P < 0.001; unpaired Student’s t-test was used for two-group comparisons. H&E: Hematoxylin and Eosin; CASA: Computer-assisted sperm analysis

Hydrocephalus is observed in some *Dnali1*^−/−^ mice (Fig. S3-S4). We further analyzed the phenotypic changes (related to male infertility) in testes and sperm of *Dnali1*^−/−^ male mice. We found that the *Dnali1*^−/−^ male mice showed no significant difference in the testis weight/body weight ratio when compared to WT male mice (Fig. 2A and B). Hematoxylin and Eosin (H&E) staining using sperm from cauda epididymides showed no obvious morphology difference between WT and *Dnali1*^−/−^ groups (Fig. 2C). Through the computer-assisted sperm analysis (CASA) system, we found that *Dnali1*^−/−^ male mice presented significantly decreased sperm motility compared to WT male mice (Fig. 2D-F). Together, these results suggest that *Dnali1* is required for normal sperm motility.

### *Dnali1* deficiency disrupts the ultrastructure of sperm flagella

**Fig. 3:**
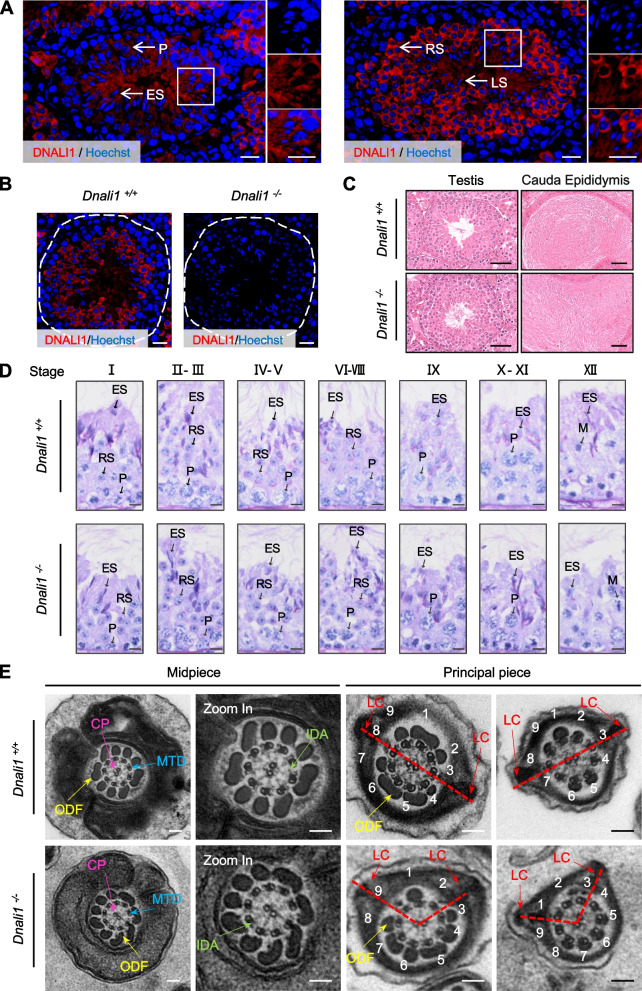
*Dnali1* deficiency disrupts the ultrastructure of sperm flagella. **A** Expression of DNALI1 during spermatogenesis. Testis sections from adult WT male mice were immunostained with an anti-DNALI1 antibody (red). Nuclei were stained with Hoechst 33342 (blue). Scale bar, 20 μm. **B** Expression analysis of DNALI1 in testes from *Dnali1*^+/+^ and *Dnali1*^−/−^ male mice. Testis sections from adult male mice were immunostained with an anti-DNALI1 antibody (red). Nuclei were stained with Hoechst 33342 (blue). Scale bar, 20 μm. **C** H&E staining of the testis and cauda epididymis obtained from *Dnali1*^+/+^ and *Dnali1*^−/−^ male mice. Scale bar, 50 μm. **D** Periodic acid–Schiff (PAS) staining of testis sections from adult *Dnali1*^+*/*+^ and *Dnali1*^*−/−*^ mice. P: pachytene spermatocyte, M: meiotic spermatocyte, RS: round spermatid, ES: elongating spermatid. Scale bar, 5 μm. (**E**) Representative TEM images of cross-sections of the midpiece and the principal piece of *Dnali1*^+/+^ and *Dnali1*^−/−^ sperm flagella. The flagella of *Dnali1*^−/−^ male mice displayed asymmetric LCs, displaying as two “protruding horns” (red dotted lines). Scale bar, 100 nm. WT, Wild-type; H&E, Hematoxylin and Eosin; TEM, Transmission electron microscopy; LC, longitudinal column (red arrows); CP, central pair of microtubules (pink arrows); ODF, outer dense fiber (yellow arrows); MTD, microtubule doublets (blue arrows); IDA, inner dynein arms (green arrows)

We next explored whether abnormalities occur in spermatogenesis. By immunofluorescence staining of testis sections from WT male mice, we observed that DNALI1 is abundant in spermatocytes, round spermatids, elongating spermatids, and late spermatids (Fig. 3A); while in *Dnali1*^−/−^ testes, no DNALI1 signal was detected in these cell types (Fig. 3B). H&E staining of sections from the testis and cauda epididymis revealed no obvious abnormalities in the sperm cells within the testicular seminiferous tubules or cauda epididymides in *Dnali1*^−/−^ male mice when compared to WT controls (Fig. 3C). Consistently, no obvious abnormalities in spermatogonia, spermatocytes, round spermatids, or elongating spermatids were observed in testicular cross-sections by Periodic acid-Schiff (PAS) staining analysis (Fig. 3D).

We further used transmission electron microscopy (TEM) to analyze sperm flagellar ultrastructure in sperm from WT and *Dnali1*^−/−^ male mice. The result showed that the midpiece and the principal piece of *Dnali1*^−/−^ flagella displayed the typical “9 + 2” microtubular structures (with visible IDAs) similar with WT controls, and peri-axonemal structures, such as ODFs and MS, were present (Fig. 3E). Immunofluorescence analysis on WT and *Dnali1*^−/−^ sperm showed the presence of DNAH2 (an IDA protein) in *Dnali1*^−/−^ sperm (Fig. S5). Intriguingly, we found that the third and eighth ODFs in the principal piece were replaced by two LCs of FSs with a symmetrical distribution in WT flagella, while the flagella of *Dnali1*^−/−^ male mice displayed asymmetric LCs, displaying as two abnormal “protruding horns” (Fig. 3E). Together, these results suggest that DNALI1 exerts functions in sperm flagellum assembly.

### *Dnali1*-associated male infertility could be overcome by intracytoplasmic sperm injection

**Fig. 4:**
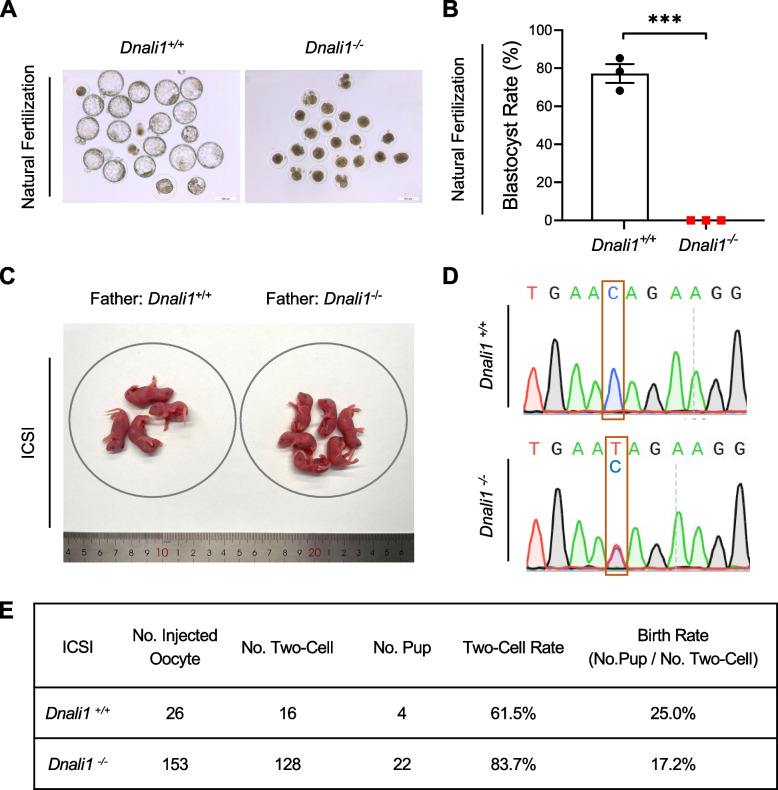
Intracytoplasmic sperm injection overcomes the infertility caused by *Dnali1* deficiency. (**A**-**B**) Representative 4.5-day blastocysts (**A**) and the blastocyst rate (**B**) to evaluate the ability of sperm in reaching oocytes for fertilization in vivo. Female WT mice were superovulated and mated with adult *Dnali1*^+/+^ and *Dnali1*^−/−^ male mice; embryos were collected from female mice at the zygote stage and then were cultured to the blastocyst stage. Scale bar, 100 μm. ***P < 0.001, unpaired Student’s *t*-test. **C** Representative offspring obtained from ICSI using sperm from *Dnali1*^+/+^ and *Dnali1*^−/−^ male mice. **D** Sequencing chromatograms of ICSI offspring from *Dnali1*^+/+^ and *Dnali1*^−/−^ male mice. **E** The rates of ICSI embryos developing into two-cell embryos and live-born pups. ICSI embryos at the two-cell stage were transferred into the oviducts of pseudopregnant female mice. WT, Wild-type; ICSI, Intracytoplasmic sperm injection

To explore the effect of *Dnali1* deficiency on the ability of sperm in reaching oocytes for fertilization in vivo, WT female mice were superovulated and respectively mated with WT or *Dnali1*^−/−^ male mice; embryos were harvested from female mice at the zygote stage and cultured in vitro to the blastocyst stage. A high rate of blastocysts was obtained in the WT group (77%), while no blastocysts were obtained for the *Dnali1*^−/−^ group (0%), indicating a failure of *Dnali1*^−/−^ sperm in reaching oocytes for fertilization in vivo (Fig. 4A and B).

To examine whether the male infertility caused by *Dnali1* deficiency could be overcome, we performed intracytoplasmic sperm injection (ICSI) using the sperm from WT and *Dnali1*^−/−^ male mice, and two-cell embryos were transferred into foster female mice. As a result, offspring were successfully obtained from *Dnali1*^−/−^ male mice; genotyping of the gDNA showed that the ICSI offspring of WT male mice were wild-type, and the ICSI offspring of *Dnali1*^−/−^ male mice were heterozygous (Fig. 4C-E). These results provide experimental evidence that male infertility caused by *Dnali1* deficiency could be overcome by the ICSI.

## Discussion

Owing to the similar spermatogenic steps and cell types between humans and mice, knockout/mutated mouse models of specific spermatogenesis-related genes are valuable tools for understanding protein functions and networks underlying male fertility, as well as for validating the genetics of human infertility [22–25]. The murine *Dnali1* gene encodes a flagellar protein that interacts with the cytoplasmic dynein heavy chain 1 [12], and DNALI1 is conserved during evolution (Fig. 1). The murine and human DNALI1 proteins share 95% identical amino acids [12], indicating that the mouse is a suitable model organism for exploring the functions of DNALI1 in spermatogenesis.

Previous studies reported that the expression levels of genes related to sperm flagellum biogenesis are significantly increased 12 days post birth [26]. Consistently, we found that *Dnali1* is abundant in the testis, and starts to express from postnatal day 15 (Fig. 1). By generating a mutated mouse model, we found that *Dnali1*^−/−^ male mice are sterile with significantly reduced sperm motility and with ultrastructural defects in the sperm flagellum (Figs. 2 and 3). The sperm flagellum is an evolutionary conserved organelle which sustains sperm motility, and is indispensable for sperm progression within the female genital tract and fertilization [9]. Functional and structural defects in the sperm flagellum often result in sperm motility abnormalities, which are characterized as asthenozoospermia in humans [27]. Previous studies identified two infertile men with asthenozoospermia harboring pathogenic mutations in *DNALI1* [14, 15]. Semen parameter analysis of the two *DNALI1*-mutated men showed significantly decreased sperm motility [14, 15]. Consistent with these phenotypes, our *Dnali1*^−/−^ male mice showed male infertility and asthenozoospermia (Fig. 1F, 2E, and F). Abnormal LC localization was observed by TEM analysis in the *DNALI1*-mutated man [15]. In *Dnali1*^−/−^ male mice, we observed ultrastructural abnormalities in the sperm flagellum, displaying as asymmetric LCs, indicating that the process of flagellum assembly was affected due to the *Dnali1* deficiency (Fig. 3).

During the draft preparation of this study, an independent study using a *Dnali1* conditional knockout (cKO) mouse model reported that DNALI1 interacts with the MEIG1/PACRG complex in the manchette, and is involved in intra-manchette transport (IMT), intra-flagellar transport (IFT) and sperm motility in mice [16]. It’s worth noting that both the reported *Dnali1* cKO male mice and our *Dnali1*^−/−^ mice display male infertility, damaged sperm motility, and disorganized fibrous sheath structure (Figs. 1F, 2E, F, 3E) [16]; these phenotypes recapitulate the reported phenotypes of the *DNALI1*-mutated infertile man [15]. The *Dnali1* cKO mice showed more severe phenotypes in terms of sperm counts and sperm gross morphology compared to our *Dnali1*^−/−^ mice, possibly owing to the different strategies used for constructing mutant mice. In addition to reproductive phenotypes, hydrocephalus was also observed in some *Dnali1*^−/−^ mice, indicating that DNALI1 may also function in motile cilia in the brain (Fig. S3).

Together, these studies collectively demonstrated *Dnali1* is indispensable for sperm motility. More importantly, our analysis of ICSI on *Dnali1*-mutated male mice achieved a good prognosis (Fig. 4). This finding is consistent with recent studies reporting good prognosis by ICSI using sperm from *DNALI1*-mutated men [14, 15]. Collectively, these studies establish that ICSI is recommended as a suitable strategy for overcoming male infertility caused by *DNALI1/Dnali1* deficiency. In addition, the consistent infertility phenotypes caused by *DNALI1/Dnali1* deficiency, and the consistent ICSI outcomes between *Dnali1*-mutated mice and *DNALI1*-mutated men, are in line with the notion that the mutant mouse model is a useful reference for analyzing the genetics of human infertility [22–25, 28, 29].

### Limitations of the study

This study does not recruit infertile men with asthenozoospermia to screen for potential mutations in human *DNALI1* gene. Future studies screening infertile men for mutations in *DNALI1* will provide more insights into the contribution of *DNALI1* deficiency to human infertility. Besides, how DNALI1 functions to facilitate the proper localization of LCs during sperm flagellum assembly needs a follow-up study.

## Conclusions

In summary, we generated a *Dnali1*-mutated mouse model and showed that *Dnali1* plays an important role in mouse spermatogenesis. *Dnali1* deficiency induced damaged sperm motility, and caused complete male infertility. In addition, *Dnali1* deficiency results in asymmetric LCs in the principal piece of sperm flagella. Finally, we show ICSI is an effective approach to overcome *Dnali1*-associated male infertility. Our study demonstrated a role of *Dnali1* in sperm flagellum assembly, sperm motility, and male fertility.

### Supplementary Information

**Additional file 1: Figure S1.** Expression analysis of *Dnali1* mRNA in the testes from *Dnali1*^*+/+*^ and *Dnali1*^*-/-*^ male mice. **Figure S2.** Immunoblotting analysis of DNALI1 protein levels in the indicated tissues of *Dnali1*^*+/+*^ and *Dnali1*^*-/-*^ mice. **Figure S3.** Hydrocephalus occurs in *Dnali1*^*-/-*^ mice. **Figure S4.** H&E staining analysis of the trachea, lung, and oviduct sections in *Dnali1*^*+/+*^ and *Dnali1*^*-/-*^ mice. **Figure S5.** Immunofluorescence analysis of DNAH2 and DNAH17 in the sperm of *Dnali1*^*+/+*^ and *Dnali1*^*-/-*^ mice. **Table S1.** Primers for gene editing, genotyping, and gene expression analysis.

## Data Availability

Data needed to evaluate the conclusions are present in the paper and the Supplementary Materials. Newly generated mutant mouse model can be provided by the corresponding author pending scientific review and a completed material transfer agreement.

## Abbreviations

CASA: Computer-assisted sperm analysis
FS: Fibrous sheath
H&E: Hematoxylin and Eosin
IDA: Inner dynein arm
ICSI: Intracytoplasmic sperm injection
IMT: Intra-manchette transport
IFT: Intra-flagellar transport
LCs: Longitudinal columns
MTD: Microtubule doublets
MS: Mitochondrial sheath
ODA: Outer dynein arm
ODF: Outer dense fiber
qPCR: Real-time quantitative PCR
WT: Wild-type
