# Supplementary material for: Dnali1 is required for sperm motility and male fertility in mice
Source: Basic Clin Androl. 2023 Nov 23;33:32. doi: 10.1186/s12610-023-00205-y (PMC10666298; doi:10.1186/s12610-023-00205-y)
Supplement: Supplementary file 1 — Additional file 1: Figure S1. Expression analysis of Dnali1 mRNA in the testes from Dnali1+/+ and Dnali1-/- male mice. Figure S2. Immunoblotting analysis of DNALI1 protein levels in the indicated tissues of Dnali1+/+ and Dnali1-/- mice. Figure S3. Hydrocephalus occurs in Dnali1-/- mice. Figure S4. H&E staining analysis of the trachea, lung, and oviduct sections in Dnali1+/+ and Dnali1-/- mice. Figure S5. Immunofluorescence analysis of DNAH2 and DNAH17 in the sperm of Dnali1+/+ and Dnali1-/- mice. Table S1. Primers for gene editing, genotyping, and gene expression analysis. [file 12610_2023_205_MOESM1_ESM.pdf]

Supplementary Materials for

Dnali1 is required for sperm motility and male fertility in mice

Yiling Zhou, Yaling Wang, Jingwen Chen, Bangguo Wu, Shuyan Tang, Feng Zhang, Chunyu Liu, Lingbo Wang

Supplementary Materials (uploaded as a separate file). This file includes:

—Supplementary File: Figures S1-S5 and Table S1

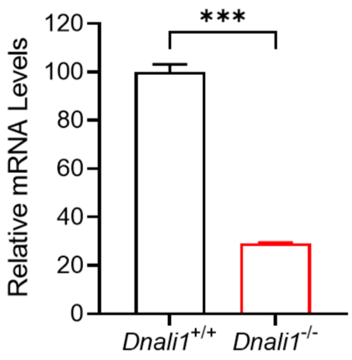

Figure S1. Expression analysis of *Dnali1* mRNA in the testes from *Dnali1*$^{+/+}$ and *Dnali1*$^{-/-}$ male mice.

qPCR was used to detect the mRNA levels of *Dnali1* in the testes of *Dnali1*$^{+/+}$ and *Dnali1*$^{-/-}$ adult male mice. ***, $P < 0.001$, unpaired Student's t-test.

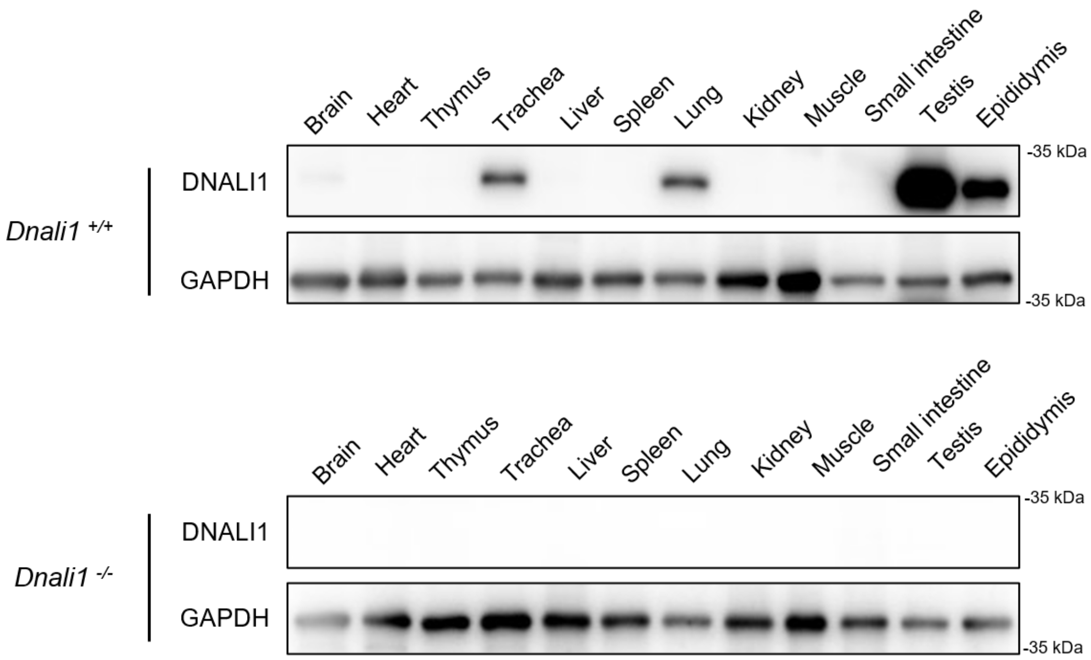

Figure S2. Immunoblotting analysis of DNALI1 protein levels in the indicated tissues of *Dnali1*$^{+/+}$ and *Dnali1*$^{-/-}$ mice.

An anti-DNALI1 antibody (17601-1-AP, Proteintech) was used for detecting DNALI1 protein levels. GAPDH was used as a loading control.

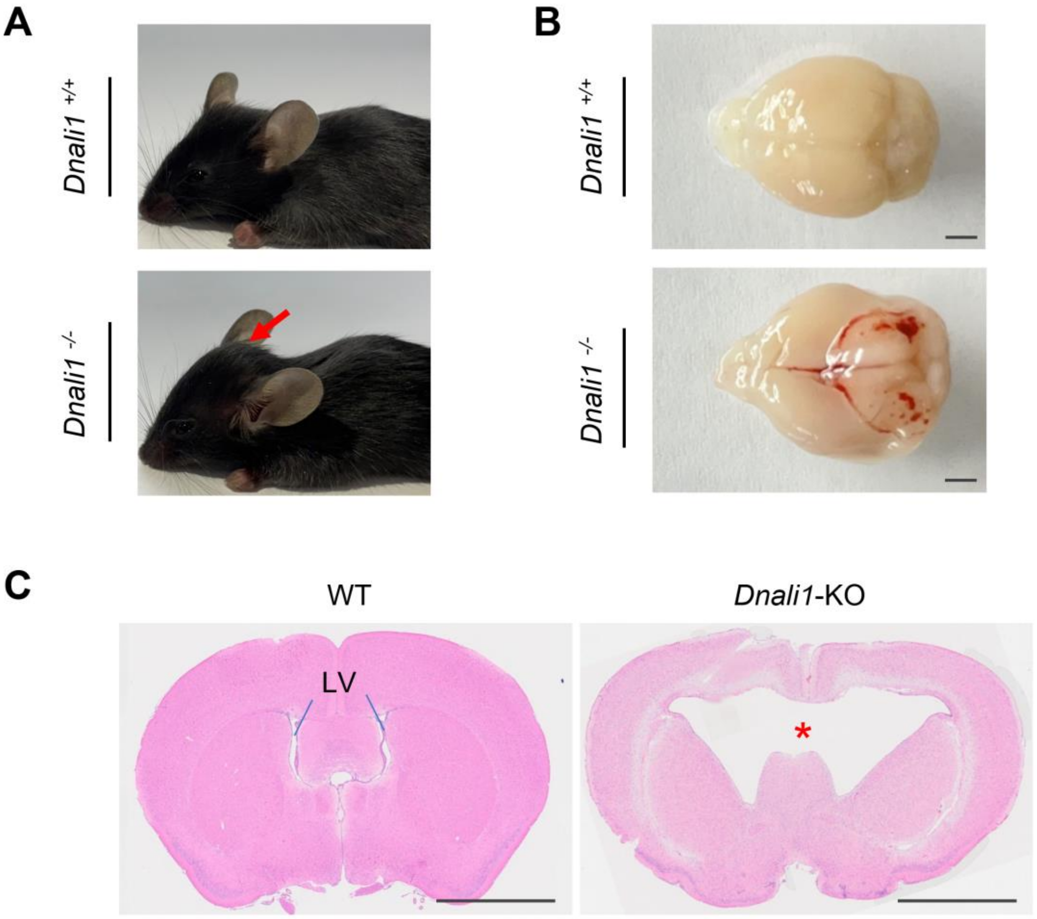

Figure S3. Hydrocephalus occurs in *Dnali1*$^{-/-}$ mice.

(A) Representative images of *Dnali1*$^{+/+}$ and *Dnali1*$^{-/-}$ mice at postnatal day 24 (P24). Red arrow indicates the enlarged and dome-shaped skull.

(B) Whole brains of *Dnali1*$^{+/+}$ and *Dnali1*$^{-/-}$ mice at P24. Scale bar, 2 mm.

(C) Coronal brain cross-sections of *Dnali1*$^{+/+}$ and *Dnali1*$^{-/-}$ mice at P24. LV, lateral ventricle. Red asterisk indicates the enlarged ventricle. Scale bar, 2 mm.

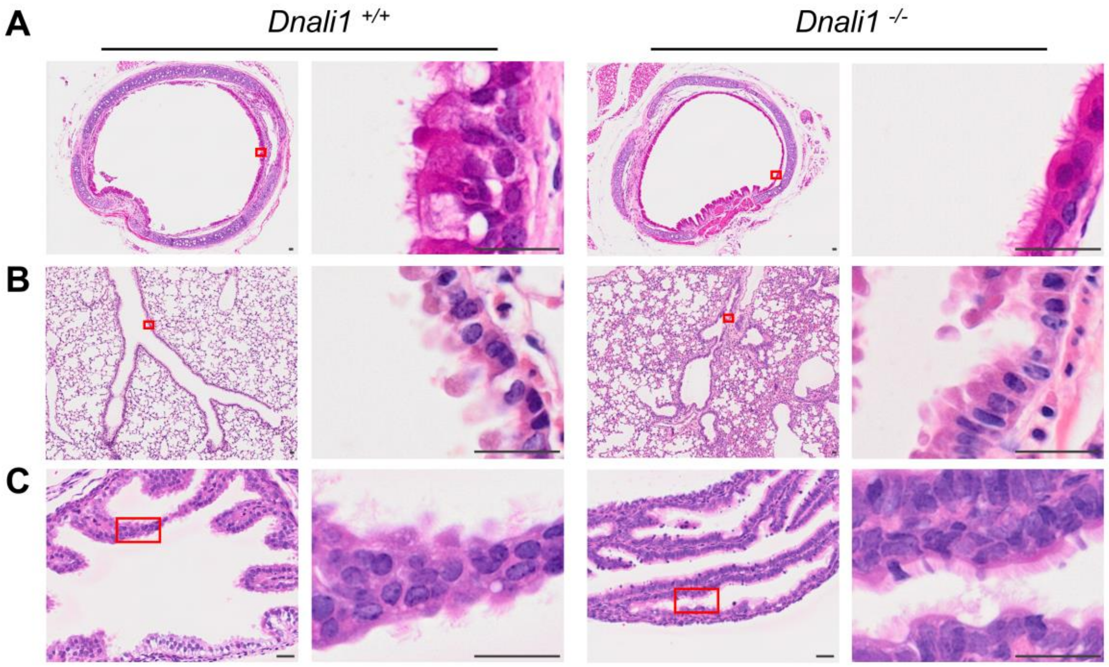

Figure S4. H&E staining analysis of the trachea, lung, and oviduct sections in *Dnali1*$^{+/+}$ and *Dnali1*$^{-/-}$ mice.

(A) H&E staining of trachea sections in *Dnali1*$^{+/+}$ and *Dnali1*$^{-/-}$ mice at 24 days old. Boxed regions on the right depict higher magnifications. Scale bar, 20 µm.

(B) H&E staining of lung sections in *Dnali1*$^{+/+}$ and *Dnali1*$^{-/-}$ mice at 24 days old. Boxed regions on the right depict higher magnifications. Scale bar, 20 µm.

(C) H&E staining of oviduct sections in *Dnali1*$^{+/+}$ and *Dnali1*$^{-/-}$ mice at 24 days old. Boxed regions on the right depict higher magnifications. Scale bar, 20 µm.

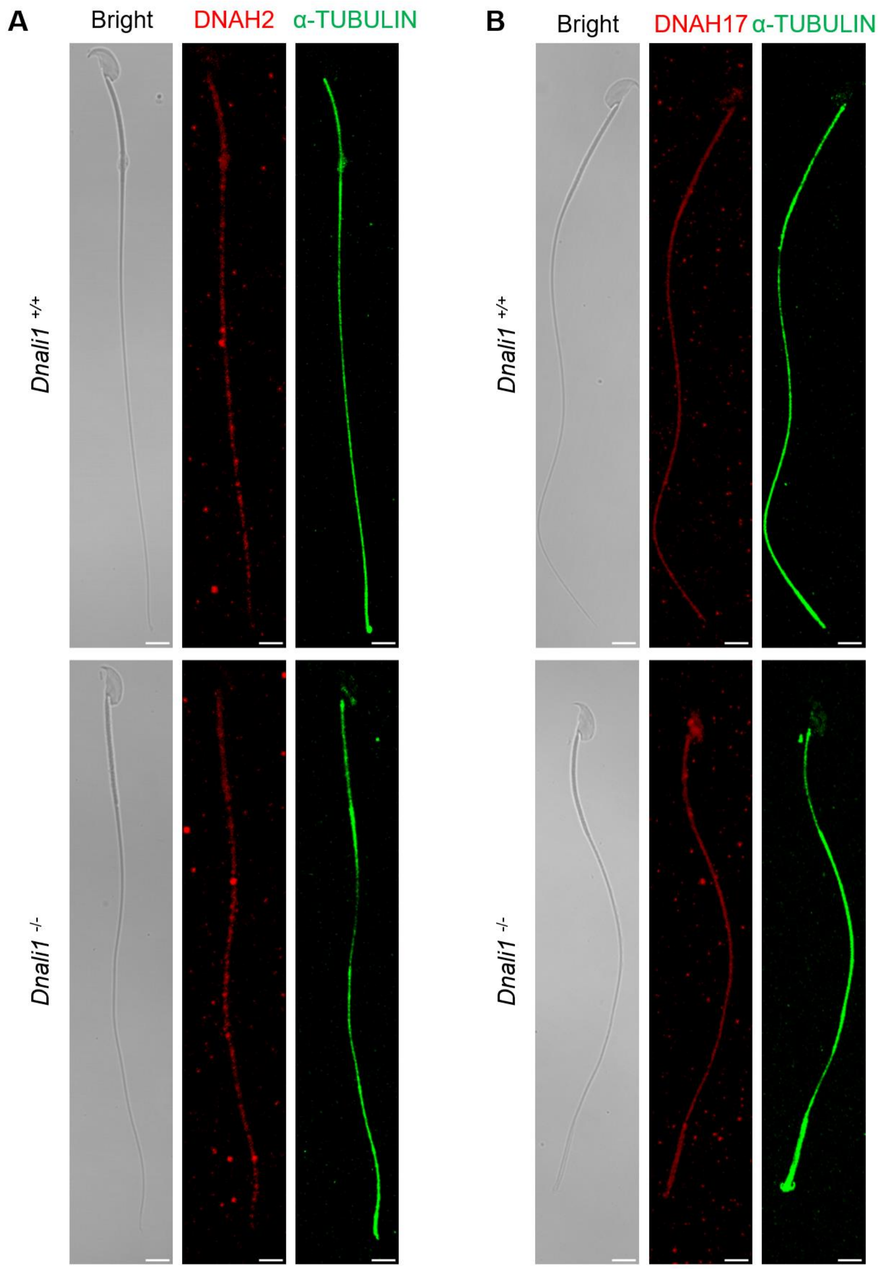

Figure S5. Immunofluorescence analysis of DNAH2 and DNAH17 in the sperm of *Dnali1*$^{+/+}$ and *Dnali1*$^{-/-}$ mice.

(A) Immunofluorescence analysis of DNAH2 in the sperm of *Dnali1*$^{+/+}$ and *Dnali1*$^{-/-}$ mice. Scale bar, 5 μm.

(B) Immunofluorescence analysis of DNAH17 in the sperm of *Dnali1*$^{+/+}$ and *Dnali1*$^{-/-}$ mice. Scale bar, 5 μm.

Table S1. Primers for gene editing, genotyping, and gene expression analysis.

Primer	Sequence (5’-3’)	Application
sg*Dnali1*-B-F	TAATACGACTCACTATAGGGTGAACAGAAG GCGAAATGCGGTTTTAGAGCTAGAAATAGC	Construction of *Dnali1*-mutated mice
sgRNA-IVT-R	AAAAGCACCGACTCGGTGCCAC	
Dnali1-OF	AGGAAAGTGAGTGGGATGACG	Genotyping of *Dnali1*-mutated mice
Dnali1-OR	GAGGATCATATCTGCCTAATG	
Dnali1-IF	ATTGCCTTAGGTCGGACTGTC	
Dnali1-IR	TGGGAAACACTCTAGGTCTGG	
Dnali1-RNA-F4	CAGCAACTGAAGGCCCAACT	Analysis of mRNA expression
Dnali1-RNA-R4	ATGAGTGGTGACCAGGGAAG	
Gapdh-RNA-F2	GTCGTGGAGTCTACTGGTGTC	
Gapdh-RNA-R2	GAGCCCTTCCACAATGCCAAA	
